# CircITGA7 regulates malignant phenotypes in bladder cancer cells via targeting miR‐330‐3p/KLF10 axis

**DOI:** 10.1002/kjm2.12821

**Published:** 2024-03-25

**Authors:** Xian‐Xu Yang, Chao Wang

**Affiliations:** ^1^ Department of Urology The First Affiliated Hospital of Jinzhou Medical University Jinzhou China

**Keywords:** bladder cancer, circITGA7, KLF10, miR‐330‐3p, miRNA sponge

## Abstract

Bladder cancer (BCa) is one of the common malignancies. Circular RNAs (circRNAs) play regulatory roles in cancer progression. CircITGA7 is a circRNA generated from several exons of ITGA7. The potential role of circITGA7 in BCa remains unknown and needs to be explored. Quantitative real time polymerase chain reaction (qRT‐PCR) was used to assess circITGA7 and miR‐330‐3p expression in BCa tissues and cell lines. Kaplan–Meier analysis was used to evaluate the overall survival of these BCa patients. The biological function of circITGA7 was examined by overexpression of circITGA7 using CCK‐8, EdU, wound‐healing, and Transwell assays. Xenograft assay was performed to further validate the in vitro results. To explore the mechanism of circITGA7, luciferase reporter, RNA pull‐down, fluorescence in situ hybridization (FISH) assays were employed to examine the binding interaction among circITGA7, miR‐330‐3p and kruppel‐like factor 10 (KLF10). Western blot was used to study the protein levels of KLF10.CircITGA7 was downregulated in BCa tissues and cell lines and indicated longer overall survival. Moreover, circITGA7 restricted cell proliferation, migration and invasion of BCa through negatively regulating miR‐330‐3p. The in vivo model showed that circITGA7 influenced the tumor growth. Besides, the overexpression of miR‐330‐3p promoted cell progression by directly targeting KLF10. Mechanistically, circITGA7 inhibited BCa progression by activating KLF10 via targeting miR‐330‐3p.CircITGA7 alleviates BCa cell progression via circITGA7/hsa‐miR‐330‐3p/KLF10 axis, which may provide novel therapeutic targets for BCa.

## 1 INTRODUCTION

Bladder cancer (BCa) is a common malignant tumor of the urinary system, with an incidence rate of 10th among malignant tumors worldwide and a rising trend year by year, seriously threatening the life and health of human beings.^1^, ^2^ Currently, the pathogenesis of BCa is still not completely understood in the clinic, and there is a lack of effective treatments, so early detection and prevention of disease progression, metastasis and recurrence are of great significance in saving patients' lives and prolonging their survival.^3^, ^4^

With the advent of high‐throughput sequencing technologies and bioinformatics analysis, the study of circular RNAs (circRNAs) has exploded in recent years, and thousands of circRNAs have been successfully identified in a variety of cell lines and different species.^5^ CircRNAs are a class of non‐coding RNA molecules that do not have a 5′ end cap and a 3′ end tail and forms a loop structure with covalent bonds.^6^ Currently, the focus of circRNAs research has shifted from initial large‐scale screening and identification to the study of the function and mechanism of specific circRNAs.^7^ Studies show that circRNAs play a key role in many diseases and may be associated with cardiovascular diseases, neurological disorders and cancers, especially as potential biomarkers for cancer diagnosis and treatment.^8^, ^9^ The biological functions of circRNAs in cancer have been predicted and investigated in a large number of experiments, and circRNAs have been found to act as molecular sponges for miRNAs, that is, endogenous competing RNAs (ceRNAs), which is the most common biological function of circRNAs.^10^ Currently, a large number of circRNAs have also been identified in BCa, and some of them have been shown to bind to related miRNAs, thereby affecting the molecular mechanisms of BCa proliferation, apoptosis, migration and invasion, and influencing the development and progression of BCa.^11^, ^12^

CircITGA7 is formed from the transcript of the ITGA7 gene, a member of the integrin adhesion molecule family on human chromosome 12 with approximately 256 bp. Low expression of circITGA7 in colorectal cancer tissues has been reported to inhibit colorectal cancer growth by regulating the RAS pathway^13^ or by acting as a “sponge” for miR‐3187‐3p^14^ and miR‐766.^15^ In addition, circITGA7 has been reported to be lowly expressed in gastric cancer^16^ and prostate^17^ cancer with oncogenic effects. In some other cancers, such as thyroid cancer,^18^ osteosarcoma,^19^ and glioma,^20^ circITGA7 expression is elevated and exerts a pro‐carcinogenic effect. A previous paper found that circITGA7 was significantly under‐expressed in BCa tissues.^21^ However, the expression level and mechanism of action of circITGA7 in BCa have not been reported in the literatures. Exploring the expression of circITGA7 in BCa and its function can provide further insight into the molecular mechanisms of BCa development and progression.

## 2 MATERIALS AND METHODS

### 2.1 Tissue samples

During January 2017 to January 2018, we collected 50 BCa samples and paired normal tissues with written informed consent. Ethics Committee of The First Affiliated Hospital of Jinzhou Medical University approved the experimental protocols.

### 2.2 Cell culture

Human BCa cell lines (5637, J82, RT4, T24, and SW780) and normal urothelial cell line SV‐HUC‐1 were purchased from American Type Culture Collection (ATCC, Manassas, VA, USA). Cells were cultured in Roswell Park Memorial Institute (RPMI) 1640 medium supplemented with 10% fetal bovine serum (FBS), 100 μg/mL streptomycin and 100 units/mL penicillin (all from Gibco, Carlsbad, CA, USA), and incubated in a humidified atmosphere at 37°C with 5% CO_2_.

### 2.3 Cell transfection

To construct the overexpression plasmid, the sequence of circITGA7 was cloned into the pcDNA 3.1(+) expression vector, using the empty vector as negative control according to the previous study.^13^ The miR‐330‐3p mimics (5′‐GCAAAGCACACGGCCUGCAGAGA‐3′) and inhibitor (anti‐miR‐330‐3p, 5′‐UCUCUGCAGGCCGUGUGCUUUGC‐3′), scrambled negative controls (miR‐NC, 5′‐UUUGUACUACACAAAAGUACUG‐3′; anti‐NC, 5′‐CAGUACUUUUGUGUAGUACAAA‐3′) were purchased from GenePharma (Shanghai, China). Small interfering RNAs (siRNAs) targeting kruppel‐like factor 10 (si‐KLF10; 5′‐GGAGCGACCATTTAACCAA‐3′) synthesized and provided by GenePharma (China) was used to silence KLF10 in T24 and 5637 cells, using si‐NC (5′‐TTCTCCGAACGTGTCACGT‐3′) as a scramble control. Cell transfection was conducted with Lipofectamine 3000 Reagent (Invitrogen, Carlsbad, CA, USA) according to the manufacturer's instruction.

### 2.4 RNA extraction and quantitative real time polymerase chain reaction

Total RNA was extracted from tissues or cultured cells via Trizol (Invitrogen, USA) and treated with DNase I (Roche Diagnostics, Indianapolis, IN, USA) to remove residual DNA. A total of 2 μg RNA was reversely transcribed to cDNA with oligo (dT) primers using reverse transcription kit (TaKaRa Biotechnology Co., Ltd, Dalian, China), and then used for quantitative PCR with SYBR Green qPCR Master Mixes (Applied Biosystems, Carlsbad, CA, USA) on ABI 7300 system according to the manufacturer's instruction. To confirm the specificity of the circITGA7 PCR products, we separated the PCR products amplified by divergent primers (forward, 5′‐TCCCCTGATAGCCACTACCT‐3′; reverse, 5′‐CGACCAATCATATCCCGCGT‐3′) on agarose gels using convergent primers (forward, 5′‐CTGACTCCATGTTCGGGATCA‐3′; reverse, 5′‐CACCTGTGAAGGTTTGGCG‐3′) as control. The oligonucleotides used as PCR primers were as follows: circITGA7 5′‐CCCCAAGGCCATGAACAATT‐3′ (F) and 5′‐TCCCCACCATCCAACTCATC‐3′ (R); miR‐330‐3p 5′‐ACACTCCAGCTGGGGCAAAGCACACGGCCTG‐3′ (F) and 5′‐TGGTGTCGTGGAGTCG‐3′ (R); KLF10 5′‐AGAAGAACCCACGGAAAT‐3′ (F) and 5′‐GAGGAAGGCACAGCAAAG‐3′ (R); GAPDH 5′‐CAAGGCTGAGAACGGGAAG‐3′ (F) and 5′‐TGAAGACGCCAGTGGACTC‐3′ (R); U6 5′‐CTCGCTTCGGCAGCACA‐3′ (F) and 5′‐AACGCTTCACGAATTTGCGT‐3′ (R). GAPDH and U6 were used for normalization. Fold changes were calculated using the 2^⁻ΔΔCt^ method.

### 2.5 Western blot

Protein lysates were extracted using radioimmunoprecipitation assay (RIPA) buffer (Thermo Fisher Scientific, Waltham, MA, USA), after which the protein samples were loaded for sodium dodecyl sulfate‐polyacrylamide gel electrophoresis followed by polyvinylidene fluoride (PVDF) membranes transferring. After blocking in Tris‐buffered saline containing 0.1% Tween‐20 (TBS‐T) with 5% nonfat dry milk for 30 min at 37°C, PVDF membranes were washed 3 times in TBS‐T and incubated with primary antibodies (Abcam, Shanghai, China) against KLF10 (ab73537) at 1/500 dilution and GAPDH (ab9485) at 1/2500 dilution overnight at 4°C. Following extensive washing, HRP‐conjugated goat anti‐rabbit polyclonal IgG (ab205718; Abcam) at a dilution of 1/3000 was used as secondary antibody. ECL Chemiluminescent Substrates (Invitrogen, USA) was applied for the visualization of immunoreactive bands, and the protein analysis was performed using ImageJ (NIH, Bethesda, MD, USA).

### 2.6 In vitro cell proliferation assays (CCK‐8 and EdU incorporation assays)

T24 and 5637 cells with different transfection were re‐seeded in 96‐well plates (2 × 10^3^ cells per well) and 10 μL CCK‐8 detection kit (Beyotime Biotechnology, Shanghai, China) per well were added at 0, 24, 48, and 72 h. The absorbance was analyzed at 450 nm after incubation at 37°C for 2 h.

Cell proliferation assays were further performed using the Cell‐Light EdU Apollo488 In Vitro Kit (Guangzhou Ruibo Biological Co., Ltd., Guangzhou, China). 150 μL of EdU medium was added to each well and incubated at 37°C for 2 h. Cells were fixed with 4% paraformaldehyde, washed with 200 μL PBS with 0.5% Triton X‐100, after which samples were incubated for 30 min in the dark by 200 μL Apollo staining solution. DAPI (1 mg/L) was added to each well and stained for 20 min in the dark, and then the cells were observed under an inverted fluorescent microscope and photographed.

### 2.7 In vitro cell migration and invasion assays (wound‐healing and Transwell assays)

Cells were seeded in triplicate into 6‐well plates for wound‐healing assay. A 200 μL plastic pipette tip was applied to the scratch wound when cells reached at a density of 90%. Images were captured by microscope (Zeiss, Germany) after 24 h, and analyzed by ImageJ (NIH, USA).

Transwell chamber (BD Biosciences, Bedford, MA, USA) with or without Matrigel coating was used to evaluate cell migration and invasion. Serum‐free media with cultured cells was added into the upper wells, while the lower chambers were filled with conditioned media with 20% FBS. The migrated or invaded cells were fixed and stained with 0.5% crystal violet and representative images were captured under microscopy at 200× magnification.

### 2.8 Bioinformatic analysis

The ENCORI database (https://starbase.sysu.edu.cn) was used to predict the putative binding sites of miR‐330‐3p in circITGA7 or KLF10.

### 2.9 Luciferase reporter assay

Luciferase reporter assays were performed to verify the direct binding between miR‐330‐3p and circITGA7 or KLF10. Briefly, wild‐type and mutant circITGA7 or KLF10 3'UTR were inserted into pmirGLO reporter vectors (Promega, Madison, WI, USA), respectively. T24 and 5637 cells were co‐transfected with miR‐330‐3p mimics and wild‐type or mutant‐type plasmids using Lipofectamine 2000 (Invitrogen, USA). Relative luciferase activity was evaluated on a dual‐luciferase reporter assay system (Promega, Madison, WI, USA) at 48 h post‐transfection. Data were expressed as the ratio of Renilla luciferase activity to firefly luciferase activity.

### 2.10 RNA pull down assay

RNA pull‐down assays were used to verify whether miR‐330‐3p was combined with circITGA7. Briefly, the biotinylated miR‐330‐3p probes containing circITGA7 binding sites were incubated with streptavidin‐coupled magnetic beads for 2 h and then added cell lysates for another 2 h. RNA complex conjugated with beads was eluted and the expression levels of circITGA7 were determined by quantitative real time polymerase chain reaction (qRT‐PCR).

### 2.11 Fluorescence in situ hybridization

Fluorescence in situ hybridization (FISH) assay was performed to determine the co‐localization of circITGA7 and miR‐330‐3p in T24 and 5637 cells using FISH Kit (RiboBio, Guangzhou, China). RiboBio (China) created the Cy3‐labeled circITGA7 and FITC‐labeled miR‐330‐3p probes. T24 and 5637 cells were fixed with 4% paraformaldehyde, and incubated with the obtained FISH probes in the hybridization buffer. DAPI was then used to stain cell nuclei. Laser confocal microscope (Leica Microsystems, Wetzlar, Germany) was used to capture images.

### 2.12 Xenograft assay

The animal protocol was approved by the Animal Care and Use Committee of The First Affiliated Hospital of Jinzhou Medical University. Female pathogen‐free BALB/c mice (4‐week‐old) were subcutaneously injected with 2 × 10^7^ T24 cells transfected with Vector or circITGA7, respectively (*n* = 6 per group). Tumor growth was monitored every 7 days. After 4 weeks, tumors were collected for real‐time PCR analysis of circITGA7 and miR‐330‐3p, and immunohistochemical (IHC) analysis of Ki‐67 and KLF10.

### 2.13 IHC staining

Tissue sections were fixed in 4% formaldehyde and embedded in paraffin. After rehydration and antigen retrieval, the slides (5 μm) were probed with anti‐Ki‐67 rabbit monoclonal antibody (Cell Signaling Technologies, USA; #9027; 1/800 dilution) and anti‐KLF10 rabbit polyclonal antibody (Boster Biological Technology, Wuhan, China; A03419‐1; 1/100 dilution) at room temperature followed by treatment with biotinylated secondary antibodies. Images were viewed under a microscope after using DAB chromogenic agents.

### 2.14 Statistical analysis

Data were expressed as mean ± standard deviation (SD). Statistical analysis was performed using SPSS 19.0 software (SPSS, Chicago, IL, USA). Correlation between circITGA7 and miR‐330‐3p expression or KLF10 level was determined via Pearson's correlation analysis. Two‐tailed student's *t*‐test was performed to compare the differences between two groups and one‐way analysis of variance (ANOVA) followed by Tukey's post hoc test was applied to compare the differences among more than two groups. *p* < 0.05 was considered statistically significant.

## 3 RESULTS

### 3.1 CircITGA7 is downregulated in BCa tissues and cells

**FIGURE 1:**
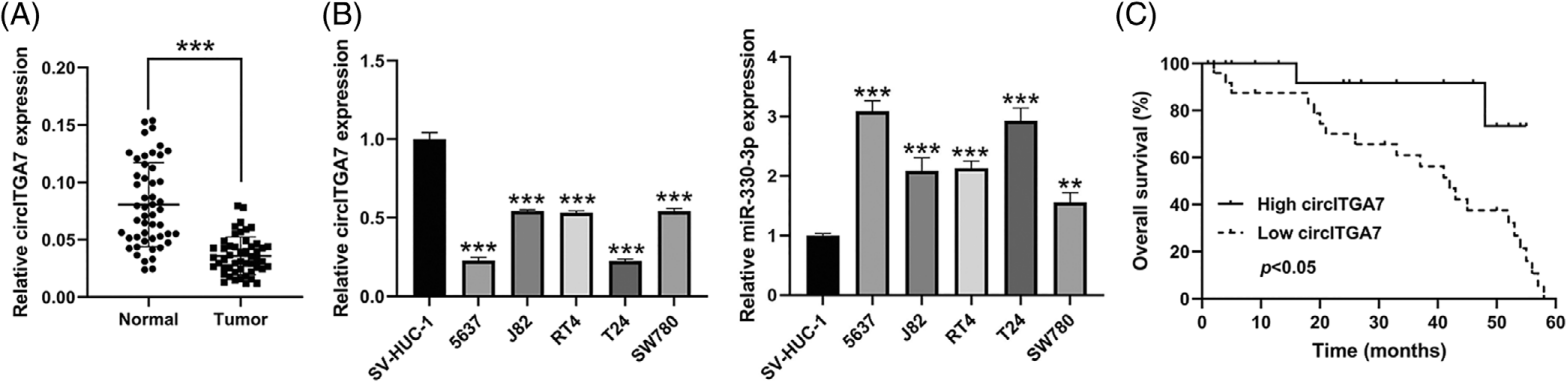
CircITGA7 is downregulated in BCa tissues and cells. (A) qRT‐PCR analysis of the expression of circITGA7 in BCa tissues and adjacent normal tissues (*n* = 50). (B) Relative expression of circITGA7 in BCa cell lines compared with normal urothelial cell line SV‐HUC‐1. Relative expression of miR‐330‐3p in BCa cell lines compared with normal urothelial cell line SV‐HUC‐1. (C) Kaplan–Meier overall survival analysis of BCa patients with high or low expression of circITGA7. **p < 0.01; ***p < 0.001 versus normal or SV‐HUC‐1.

The data of qRT‐PCR showed that the relative level of circITGA7 was significantly decreased in BCa tissues compared to the normal tissues (Figure 1A). The results also demonstrated the downregulation of circITGA7 in five human BCa cell lines, especially in T24 and 5637 cells (Figure 1B), therefore these two cell lines were selected for the following cell biology experiments. Thereafter, we also observed increased expression of miR‐330‐3p in BCa cell lines relative to the normal SV‐HUC‐1 cells. As demonstrated by Kaplan–Meier analysis, high circITGA7 expression in BCa tissues predicted a better overall survival (Figure 1C).

### 3.2 CircITGA7 overexpression restrains BCa cell proliferation, migration, and invasion in vitro

**FIGURE 2:**
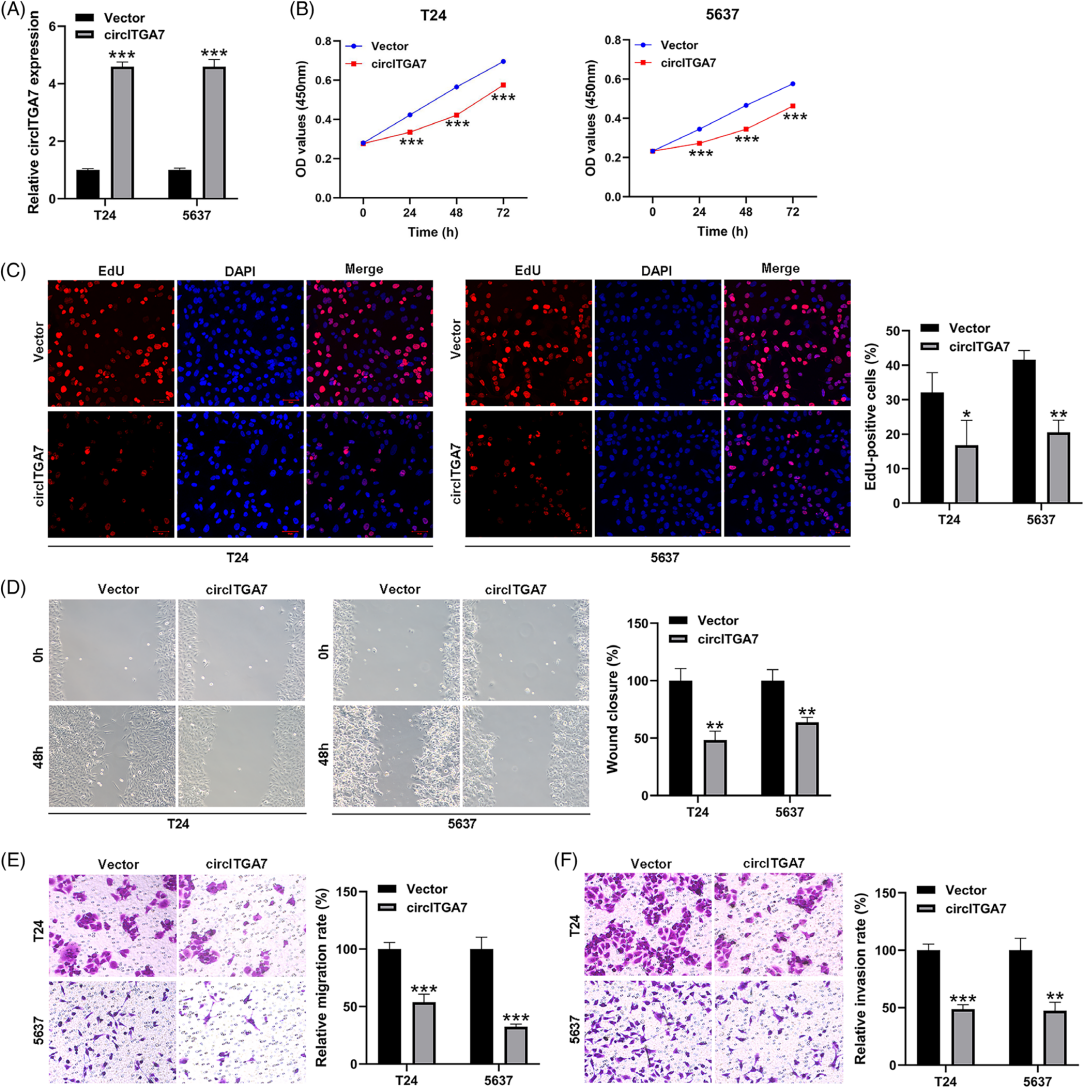
CircITGA7 overexpression restrains BCa cell proliferation, migration, and invasion in vitro. (A) The efficiency of circITGA7 overexpression vector in T24 and 5637 cell lines. (B) CCK‐8 assays in T24 and 5637 cell lines. (C) EdU assays in T24 and 5637 cell lines; blue, DAPI‐stained nuclei; green, Hoechst‐positive nuclei. (D) Wound‐healing assays in T24 and 5637 cell lines. (E, F) Transwell migration, and invasion assays in T24 and 5637 cell lines. *p < 0.05; **p < 0.01; ***p < 0.001 versus vector.

The transfection efficiency of the overexpression vector of circITGA7 was verified in T24 and 5637 cell lines (Figure 2A). CCK‐8 (Figure 2B) and EdU assays (Figure 2C) showed that the proliferation abilities in T24 and 5637 cells were significantly decreased by the upregulation of circITGA7. To further investigate the impact of the overexpression of circITGA7 on the migration capacities of T24 and 5637 cells, wound‐healing (Figure 2D) and Transwell assays (Figure 2E) were performed, which demonstrated that the migratory cells were observably reduced after circITGA7 upregulation. Transwell assays also suggested that circITGA7 overexpression reduced the cell invasion (Figure 2F). Our results demonstrated that the upregulation of circITGA7 significantly suppressed the proliferation, migration and invasion abilities of T24 and 5637 cell lines.

### 3.3 CircITGA7 overexpression attenuates the tumor growth of BCa in vivo

**FIGURE 3:**
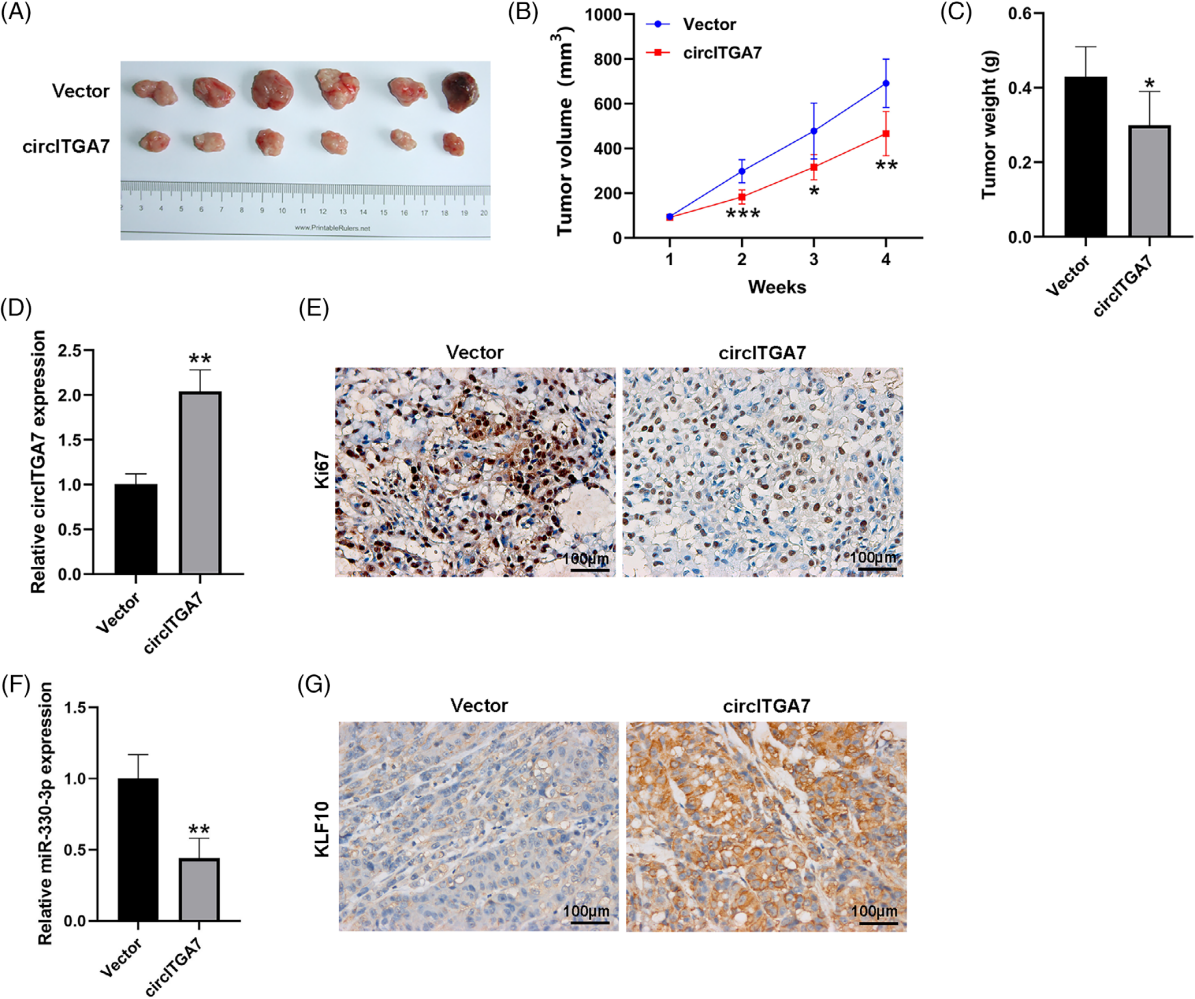
CircITGA7 overexpression attenuates the tumor growth of BCa in vivo. T24 cells stably overexpressing circITGA7 were injected subcutaneously into BALB/c nude mice (*n* = 6 per group). (A) Representative images of xenograft tumors. (B, C) Tumor volume and weight of the resected tumors. (D) qRT‐PCR analysis of the expression of circITGA7 in the resected tumors. (E) IHC analysis for Ki‐67 in resected tumors; scale bars = 100 μm. (F) qRT‐PCR analysis of the expression of miR‐330‐3p in the resected tumors. (G) IHC analysis for KLF10 in resected tumors; scale bars = 100 μm. *p < 0.05; **p < 0.01; ***p < 0.001 versus vector.

To validate the oncogenicity of circITGA7 in vivo, the xenograft animal model was constructed through injecting T24 cells stably overexpressing circITGA7 into nude mice. Our results demonstrated that the tumor size and weight were significantly reduced in the circITGA7‐overexpressed mice compared to the control group (Figure 3A–C). The expression of circITGA7 was upregulated in xenograft tissues in the circITGA7 group with respect to the control group (Figure 3D). Also, the number of Ki67‐positive cells was fewer in the circITGA7 group than that in the control group (Figure 3E). Our results demonstrated that the overexpression of circITGA7 inhibited tumor proliferation in vivo. Meanwhile, the levels of miR‐330‐3p were significantly decreased (Figure 3F), while KLF10 was increased (Figure 3G) in xenograft tumors of circITGA7‐overepressing group.

### 3.4 MiR‐330‐3p is sponged by circITGA7 in BCa

**FIGURE 4:**
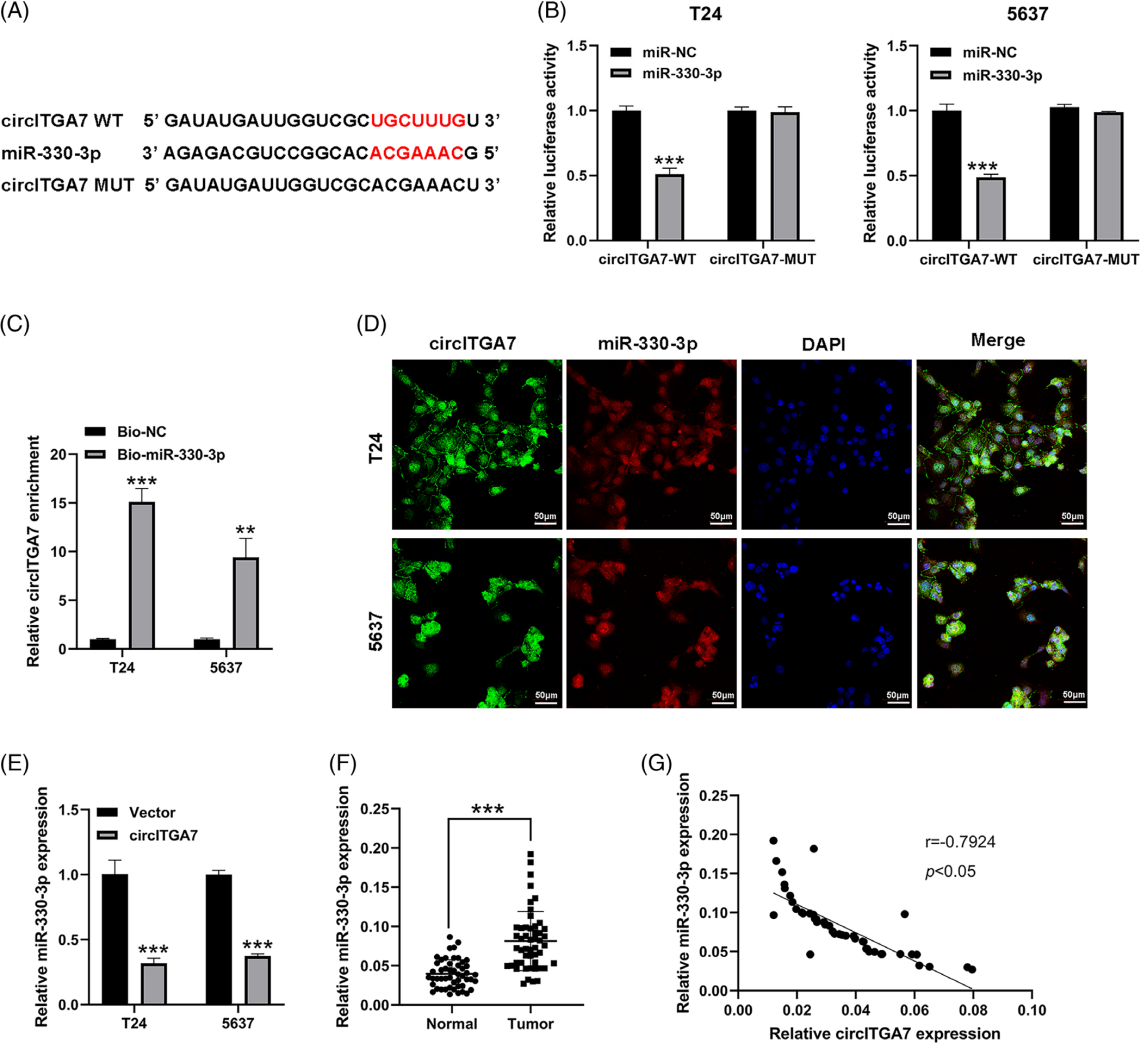
MiR‐330‐3p is sponged by circITGA7 in BCa. (A) Potential binding sequences of miR‐330‐3p and circITGA7. (B) Dual luciferase reporter assays between circITGA7 and miR‐330‐3p in T24 and 5637 cell lines. ***p < 0.001 versus miR‐NC. (C) RNA pull‐down analysis of circITGA7 and miR‐330‐3p in T24 and 5637 cell lines. **p < 0.01; ***p < 0.001 versus Bio‐NC. (D) FISH for colocalization of endogenous circITGA7 and miR‐330‐3p in T24 and 5637 cell lines; scale bars = 20 μm. (E) The expression level of miR‐330‐3p in T24 and 5637 cell lines transfected with circITGA7. ***p < 0.001 versus vector. (F) The expression level of miR‐330‐3p in BCa tissues. ***p < 0.001 versus normal. (G) Pearson correlation analysis of miR‐330‐3p and circITGA7 in BCa tumor samples. *p < 0.05.

Based on the bioinformatics analysis of ENCORI database, miR‐330‐3p was predicted as the putative downstream miRNA that could bind to circITGA7 (Figure 4A). The dual‐luciferase reporter assay showed that the luciferase activities of circITGA7 wild plasmids were notably restrained by upregulating miR‐330‐3p (Figure 4B). Additionally, the interaction of circITGA7 with miR‐330‐3p was verified by RNA pull‐down assay (Figure 4C). FISH assay also confirmed that circITGA7 co‐localized with miR‐330‐3p in the cytoplasm of T24 and 5637 cells (Figure 4D). Figure 4E showed that miR‐330‐3p was silenced by circITGA7 upregulation. The expression of miR‐330‐3p was found to be significantly upregulated in BCa tissues compared to the normal tissues (Figure 4F), and its expression level was negatively associated with circITGA7 expression in BCa tissues (Figure 4G). Our results demonstrated that circITGA7 acted as a sponge of miR‐330‐3p.

### 3.5 MiR‐330‐3p regulates the oncogenic properties of BCa cells, and targets KLF10

**FIGURE 5:**
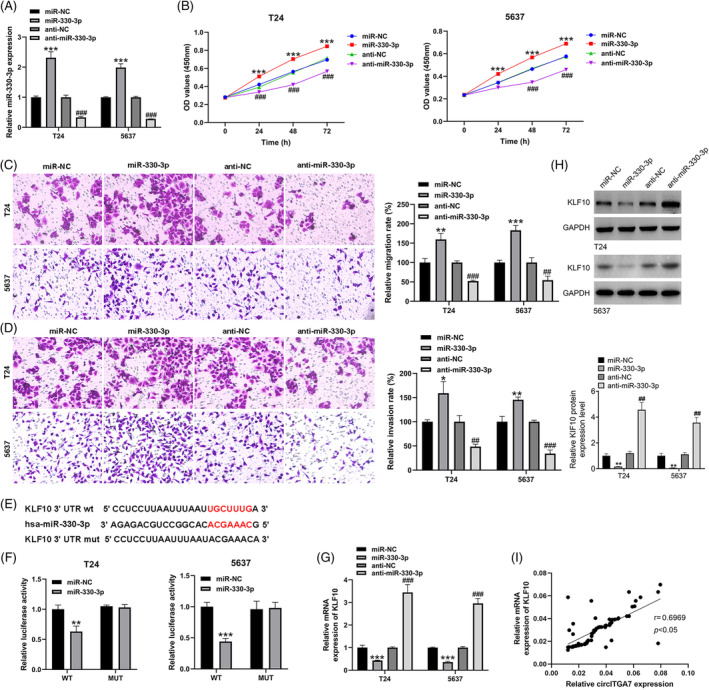
MiR‐330‐3p regulates the oncogenic properties of BCa cells, and targets KLF10. (A) The transfection efficiencies of miR‐330‐3p mimics and inhibitors in T24 and 5637 cells. (B) CCK‐8 assays in T24 and 5637 cell lines. (C, D) Transwell migration, and invasion assays in T24 and 5637 cell lines. (E) Potential binding sequences of miR‐330‐3p and KLF10. (F) Dual luciferase reporter assays between miR‐330‐3p and KLF10 in T24 and 5637 cell lines. (G, H) KLF10 mRNA and protein levels in T24 and 5637 cell lines transfected with miR‐330‐3p mimics and inhibitors. *p < 0.05; **p < 0.01; ***p < 0.001 versus. miR‐NC. ^##^ *p* < 0.01, ^###^ *p* < 0.001 versus anti‐NC. (I) Pearson correlation analysis of KLF10 mRNA and circITGA7 in BCa tumor samples (*p < 0.05).

The transfection efficiencies of miR‐330‐3p mimics and inhibitors were evaluated in T24 and 5637 cells (Figure 5A). To investigate the proliferative potentials of miR‐330‐3p, CCK‐8 assays were performed in T24 and 5637 cell lines, which implied that miR‐330‐3p promoted the growth of BCa cells (Figure 5B). Meanwhile, results from Transwell assays showed that miR‐330‐3p could facilitate the migration (Figure 5C) and invasion (Figure 5D) abilities of T24 and 5637 cell lines. Based on the ENCORI database (Figure 5E), KLF10 was a putative target of miR‐330‐3p, and thus we tried to examine the relationship of the miR‐330‐3p and KLF10. Additionally, dual luciferase reporter assays in T24 and 5637 cell lines suggested that the relative luciferase activities were predominantly inhibited after co‐transfection with miR‐330‐3p mimics and the KLF10 3'UTR‐WT vector compared with that of cells cotransfected with the KLF10 3'UTR‐Mut vector (Figure 5F). The results of qRT‐PCR (Figure 5G) and western blotting (Figure 5H) showed that miR‐330‐3p overexpression could decrease the expression of KLF10, while miR‐330‐3p knockdown led to the increase of KLF10 level. Additionally, KLF10 was positively correlated with circITGA7 expression in BCa tissues (Figure 5I). Our results demonstrated that miR‐330‐3p decreased the expression of KLF10 and mediated the BCa cell malignant phenotypes.

### 3.6 CircITGA7 exerts the anti‐oncogenic function in BCa cells via the regulation of miR‐330‐3p/KLF10

**FIGURE 6:**
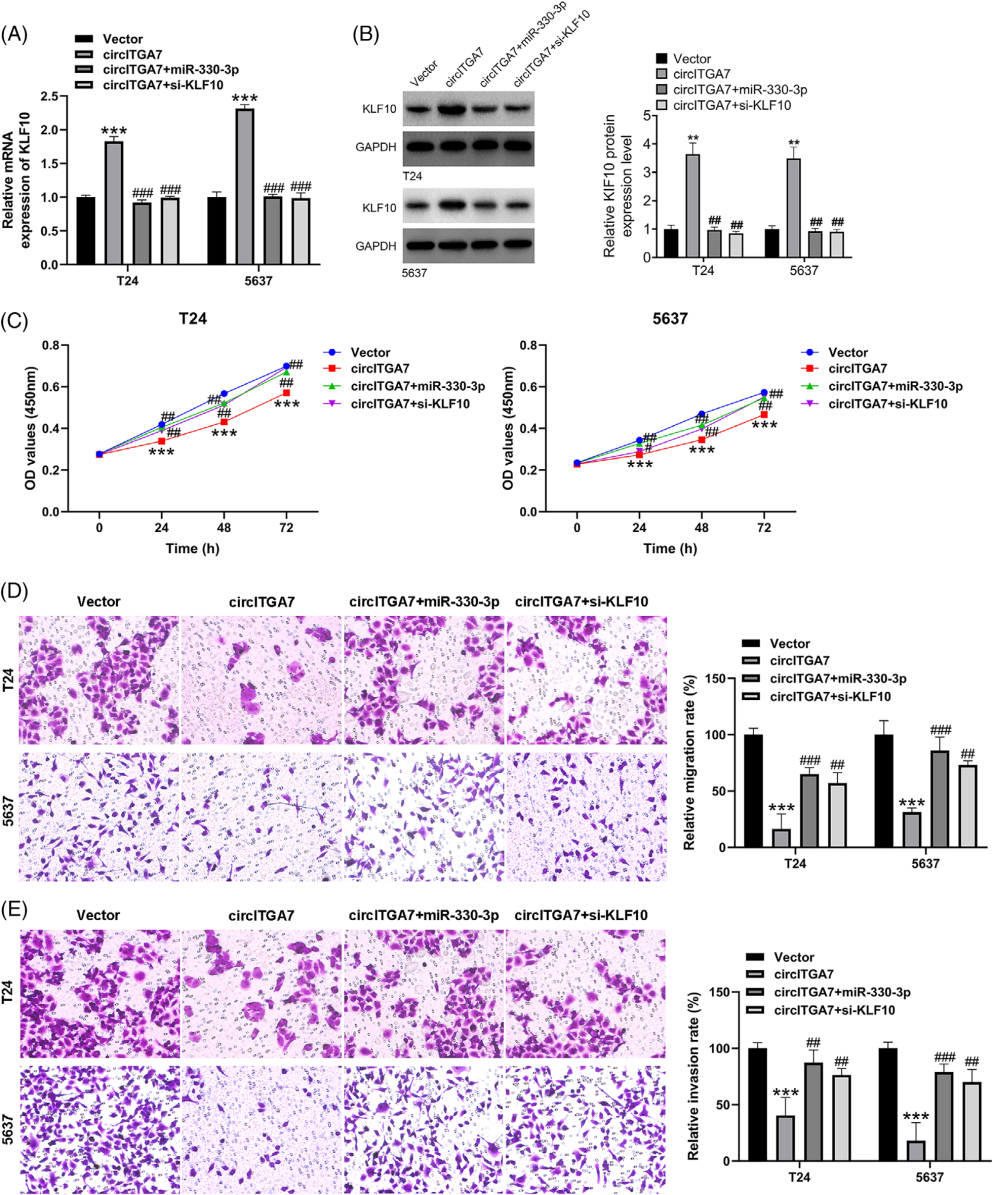
CircITGA7 exerts the anti‐oncogenic function in BCa cells via the regulation of miR‐330‐3p/KLF10. (A, B) The mRNA and protein expression level of KLF10 in T24 and 5637 cell lines after transfection by circITGA7 overexpression vector and miR‐330‐3p mimics or siKLF10. (C) Effect of circITGA7 and miR‐330‐3p mimics or siKLF10 on the proliferation in T24 and 5637 cell lines by CCK‐8 assay. (D, E) Transwell assays revealed the migration and invasion of T24 and 5637 cell lines after transfection by circITGA7 overexpression vector and miR‐330‐3p mimics or siKLF10. **p < 0.01; ***p < 0.001 versus vector. ^##^ *p* < 0.01; ^###^ *p* < 0.001 versus circITGA7.

To verify the biological function of the circITGA7/miR‐330‐3p/KLF10 axis in BCa cells, we performed the rescue assays in T24 and 5637 cell lines. Of note, KLF10 were successfully upregulated by circITGA7 overexpression, while the increased level of KLF10 induced by circITGA7 can be restrained by miR‐330‐3p mimics or KLF10 siRNA, as confirmed by qRT‐PCR (Figure 6A) and western blotting (Figure 6B). Besides, circITGA7‐induced suppressive effects on cell viability (Figure 6C), migratory ability (Figure 6D) and invasive capability (Figure 6E) could be overturned by miR‐330‐3p upregulation or the silencing of KLF10. Our results demonstrated that circITGA7 ameliorated the malignant progression in BCa via circITGA7‐miR‐330‐3p‐KLF10 axis.

## 4 DISCUSSION

BCa is one of the most common malignancies of the urinary tract, and its high recurrence and lethality remain an urgent problem despite the fact that current first‐line therapeutic regimens have improved the prognosis of patients.^22^ The pathogenesis of BCa has not been fully elucidated. As a popular research topic in the field of non‐coding RNAs, circRNAs started late compared to miRNAs and lncRNAs, but their role in tumors and other diseases cannot be overlooked.^23^, ^24^ Studies have shown that circRNA expression is dysregulated in BCa and plays an important role in BCa carcinogenesis.^11^, ^25^, ^26^ For instance, a recent study found that circSETD3 was significantly downregulated in BCa tissues and was associated with tumor size, pathological staging, lymphatic metastasis, and unfavorable prognosis.^27^ In another study, circSHPRH was downregulated in BCa tissues and cell lines and lower circSHPRH expression was associated with tumor grade, pathological stage, lymphatic metastasis and unfavorable prognosis.^28^ Another BCa‐related circRNA, circSOBP was found to be reduced in BCa tissues and cell lines, and circSOBP overexpression inhibited proliferation, migration, and invasion in vitro and tumorigenesis in vivo.^29^ Due to the biological stability of circRNA, it is expected to be a novel marker for tumor diagnosis and a therapeutic target. In this study, we discovered that circITGA7 was underexpressed in BCa and that high expression of circITGA7 was negatively associated with tumor proliferation, invasion, metastasis and unsatisfied prognosis. In vivo studies further showed that increased circITGA7 levels inhibited BCa tumorigenesis.

Functionally, circRNA molecules contain miRNA‐binding sites that act as miRNA sponges, thereby reducing miRNA repression of target genes and increasing the expression level of target genes, a mechanism of action known as the ceRNA mechanism.^30^ For example, circPPP1CB regulates the expression of SMG1 by acting as a “molecular sponge” for miR‐1307‐3p, which thereby exerting an anticancer effect in BCa.^31^ Similarly, circKDM4C, acted as a miR‐200bc‐3p sponge, and downregulated the expression of miR‐200bc‐3p, thereby deregulating its inhibition of ZEB1 and promoting the migration and invasion of BCa cells.^32^ Lin et al indicated that circFLNA, as a ceRNA, could adsorb miR‐216a‐3p, thereby indirectly regulating BTG2 and inhibiting BCa tumorigenesis as well as tumor stemness progression.^33^ Therefore, we predicted potential target miRNAs for circITGA7 by bioinformatic analysis. We found a sponging interaction between circITGA7 and miR‐330‐3p by dual luciferase reporter assay, pull‐down assay and FISH assay. The tumorigenic effects of miR‐330‐3p have been studied in multiple cancers.^34^ A previous study found that miR‐330‐3p promoted the growth and migration of BCa.^35^ In our study, we verified the biological function of miR‐330‐3p in T24 and 5637 cell lines, which was consistent with the above finding. Rescue experiments indicated that circITGA7‐induced reduction in cell proliferation, migration and invasion could be restored by miR‐330‐3p mimics, suggesting that circITGA7 achieved its anti‐oncogene function by targeting miR‐330‐3p. However, the mechanism underlying the circITGA7/miR‐330‐3p axis is still unknown and needs further investigation.

Based on previous studies, it is generally accepted that KLF10 is a tumor suppressor in a variety of cancers, such as pancreatic cancer,^36^ gastric cancer,^37^ and melanoma.^38^ In BCa, KLF10 has been proved to inhibit the tumor cell proliferation, migration and invasion.^39^, ^40^ In our study, we demonstrated the regulatory relationship between miR‐330‐3p and KLF10. Rescue experiments indicated that KLF10 inhibition abolished the action of circITGA7 on BCa cell oncogenic phenotypes. Therefore, circITGA7 influences the regulation of KLF10 through sponging miR‐330‐3p, thus regulating cell biological functions of BCa. However, although some interesting results were found in this study, there are still some limitations. First, a larger cohort of the sample sizes is essential to verify the clinical value of circITGA7 in BCa. Besides, we will further explore other possible mechanisms of miR‐330‐3p upregulation in BCa. Finally, the direct interaction between miR‐330‐3p and circITGA7 still needs a further study.

In conclusion, our study identified a BCa‐associated circRNA, circITGA7, and uncovered a novel anti‐cancer mechanism by which circITGA7 competitively interacts with miR‐330‐3p, subsequently leading to upregulation of KLF10 and inhibiting the growth and metastasis of BCa. Thus, circITGA7 has the potential to be a therapeutic target and prognostic biomarker for BCa patients.

## CONFLICT OF INTEREST STATEMENT

All authors declare no conflict of interest.

## ETHICS STATEMENT

The protocols used in this study were approved by the Ethical Review Committees of The First Affiliated Hospital of Jinzhou Medical University, and written informed consent was provided from all the patients. The animal studies were authorized by the Animal Ethic Review Committees of The First Affiliated Hospital of Jinzhou Medical University.
